# Supplementary material for: The relationships of family income and caste-status with religiousness: Mediation role of intolerance of uncertainty
Source: PLoS One. 2022 Aug 26;17(8):e0273174. doi: 10.1371/journal.pone.0273174 (PMC9417042; doi:10.1371/journal.pone.0273174)
Supplement: S3 Fig — IUP = prospective intolerance of uncertainty, IUI = inhibitive intolerance of uncertainty, GC = general castes, OBC = other backward castes, SC = scheduled castes, th. = thousand, LQ = laterality quotient (handedness). (DOCX) [file pone.0273174.s003.docx]

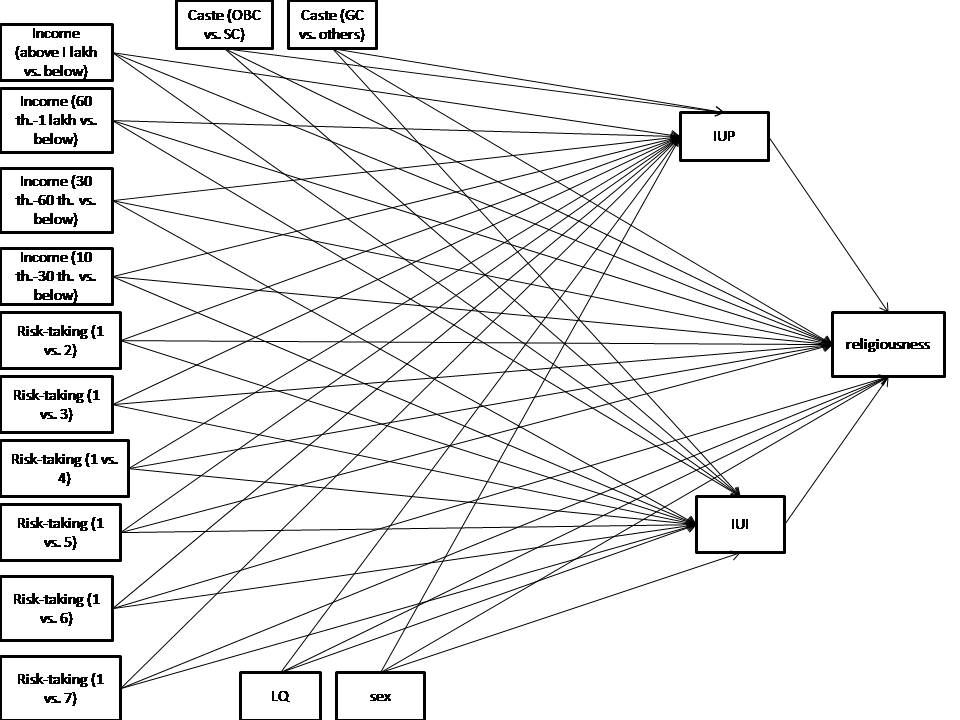


*S3 Figure*. The conceptual path diagram of the studied relationships of antecedents with IUP, IUI, and religiousness in the community sample. IUP = prospective intolerance of uncertainty, IUI = inhibitive intolerance of uncertainty, GC = general castes, OBC = other backward castes, SC = scheduled castes, th. = thousand, LQ = laterality quotient (handedness)
