# Supplementary material for: The relationships of family income and caste-status with religiousness: Mediation role of intolerance of uncertainty
Source: PLoS One. 2022 Aug 26;17(8):e0273174. doi: 10.1371/journal.pone.0273174 (PMC9417042; doi:10.1371/journal.pone.0273174)
Supplement: Attachment — Submitted filename: Response to comments of the editor.docx [file pone.0273174.s007.docx]

Response to comments of the editor

The Editor: I only see section titles for Abstract, Method and Discussion. Please include tittles for other sections.

**The Authors**: Done. Please see the marked changes in the manuscript.
